# Supplementary material for: Improving the Accuracy of Saffron Adulteration Classification and Quantification through Data Fusion of Thin-Layer Chromatography Imaging and Raman Spectral Analysis
Source: Foods. 2023 Jun 9;12(12):2322. doi: 10.3390/foods12122322 (PMC10297485; doi:10.3390/foods12122322)
Supplement: Supplementary file 1 [file foods-12-02322-s001.zip › foods-2412740-supplementary.pptx]

## Slide 1
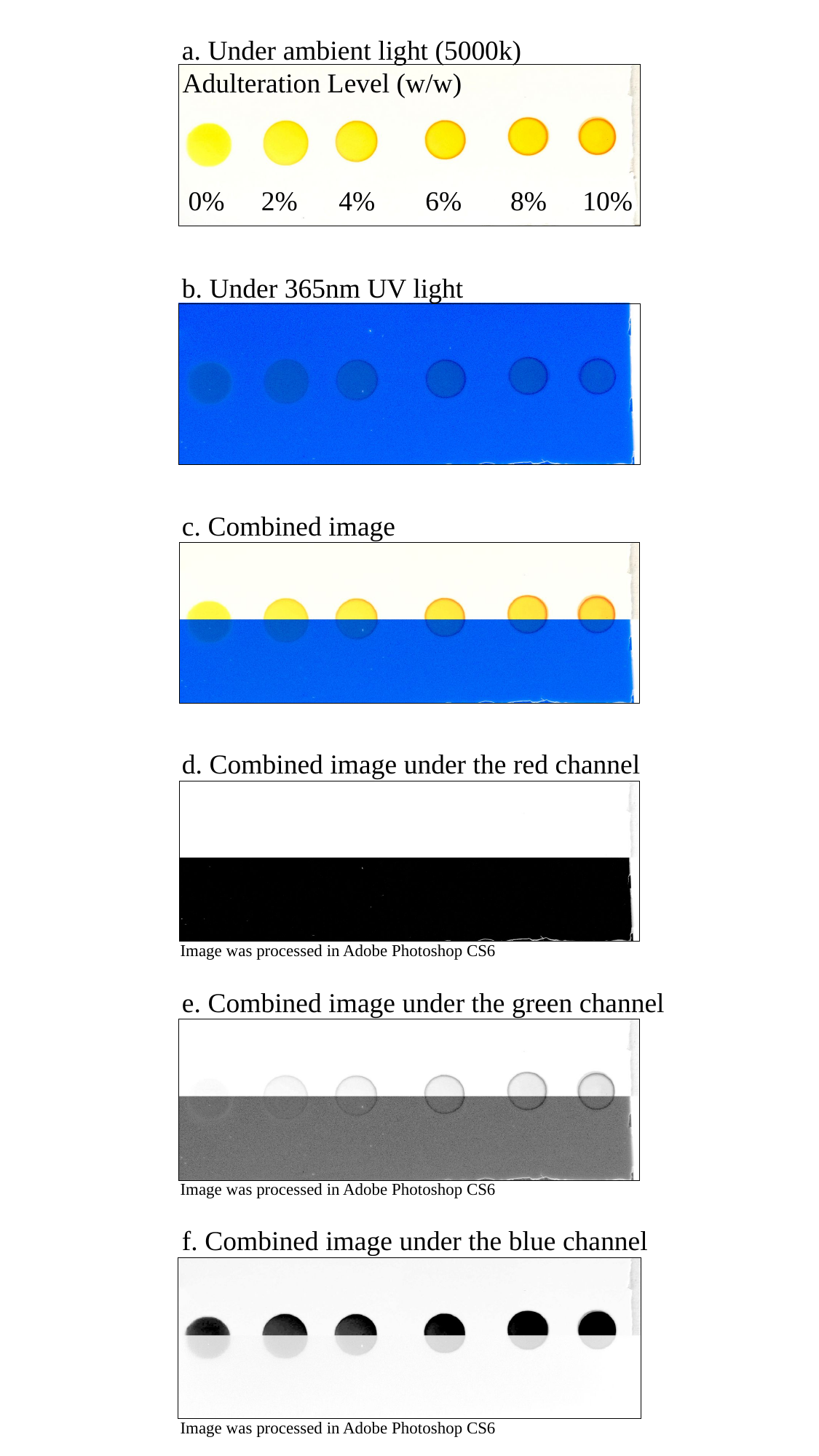

a. Under ambient light (5000k)
Adulteration Level (w/w)
0%
2%
4%
6%
8%
10%
b. Under 365nm UV light
c. Combined image
d. Combined image under the red channel
Image was processed in Adobe Photoshop CS6
e. Combined image under the green channel
Image was processed in Adobe Photoshop CS6
f. Combined image under the blue channel
Image was processed in Adobe Photoshop CS6

## Slide 2
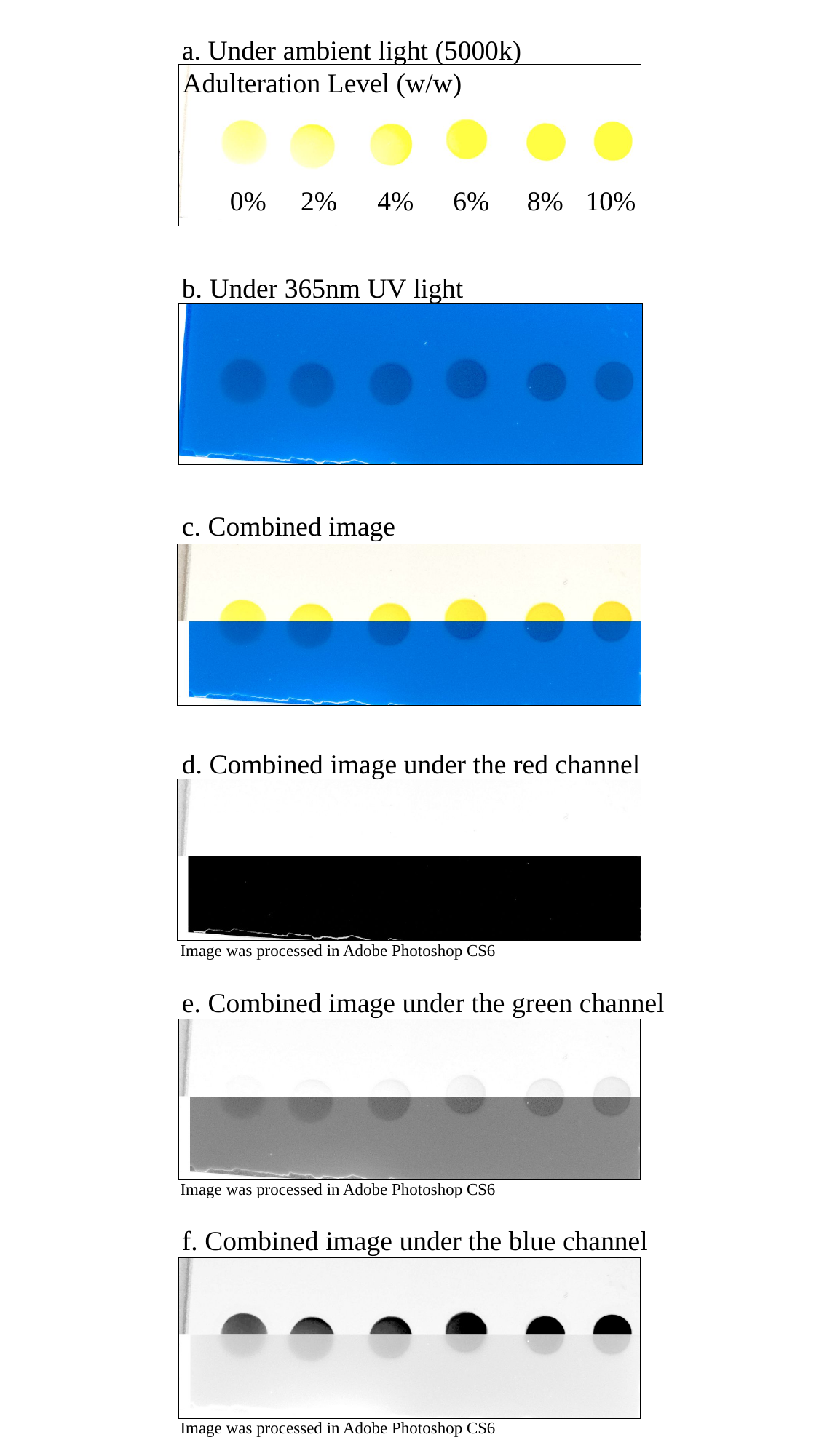

a. Under ambient light (5000k)
Adulteration Level (w/w)
0%
2%
4%
6%
8%
10%
b. Under 365nm UV light
c. Combined image
d. Combined image under the red channel
Image was processed in Adobe Photoshop CS6
e. Combined image under the green channel
Image was processed in Adobe Photoshop CS6
f. Combined image under the blue channel
Image was processed in Adobe Photoshop CS6

## Slide 3
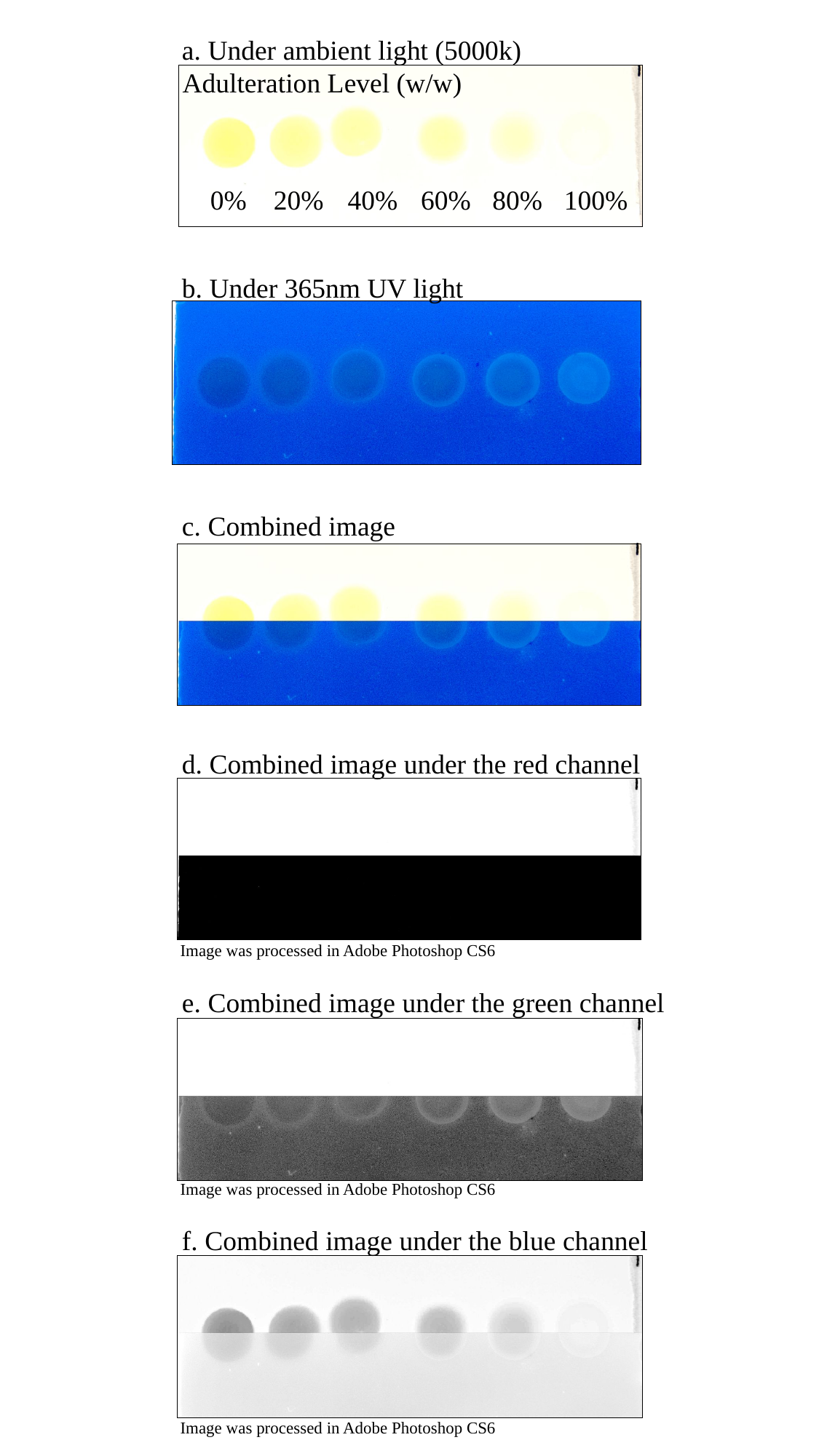

a. Under ambient light (5000k)
Adulteration Level (w/w)
0%
20%
40%
60%
80%
100%
b. Under 365nm UV light
c. Combined image
d. Combined image under the red channel
Image was processed in Adobe Photoshop CS6
e. Combined image under the green channel
Image was processed in Adobe Photoshop CS6
f. Combined image under the blue channel
Image was processed in Adobe Photoshop CS6

## Slide 4
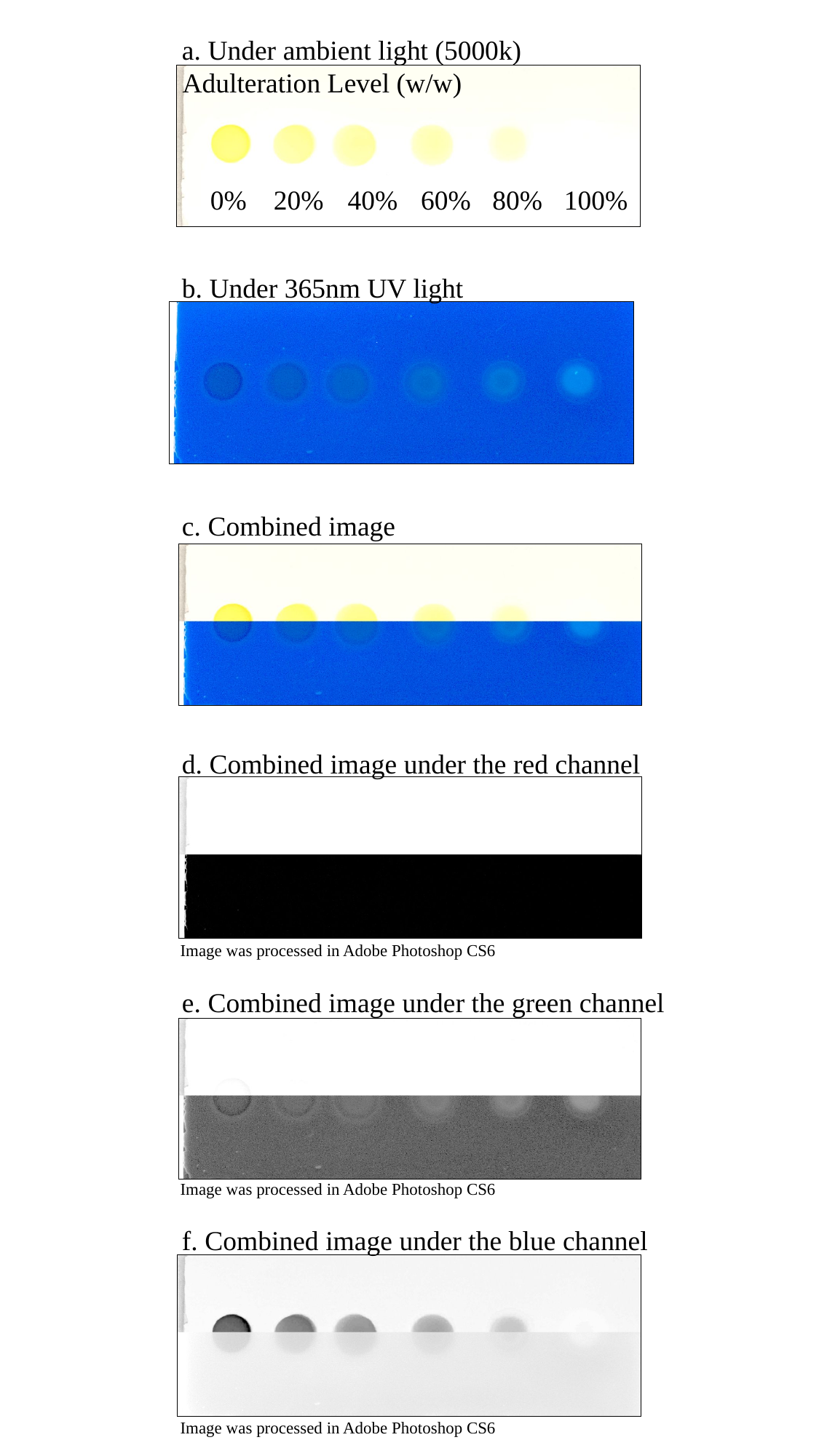

a. Under ambient light (5000k)
Adulteration Level (w/w)
0%
20%
40%
60%
80%
100%
b. Under 365nm UV light
c. Combined image
d. Combined image under the red channel
Image was processed in Adobe Photoshop CS6
e. Combined image under the green channel
Image was processed in Adobe Photoshop CS6
f. Combined image under the blue channel
Image was processed in Adobe Photoshop CS6

## Slide 5
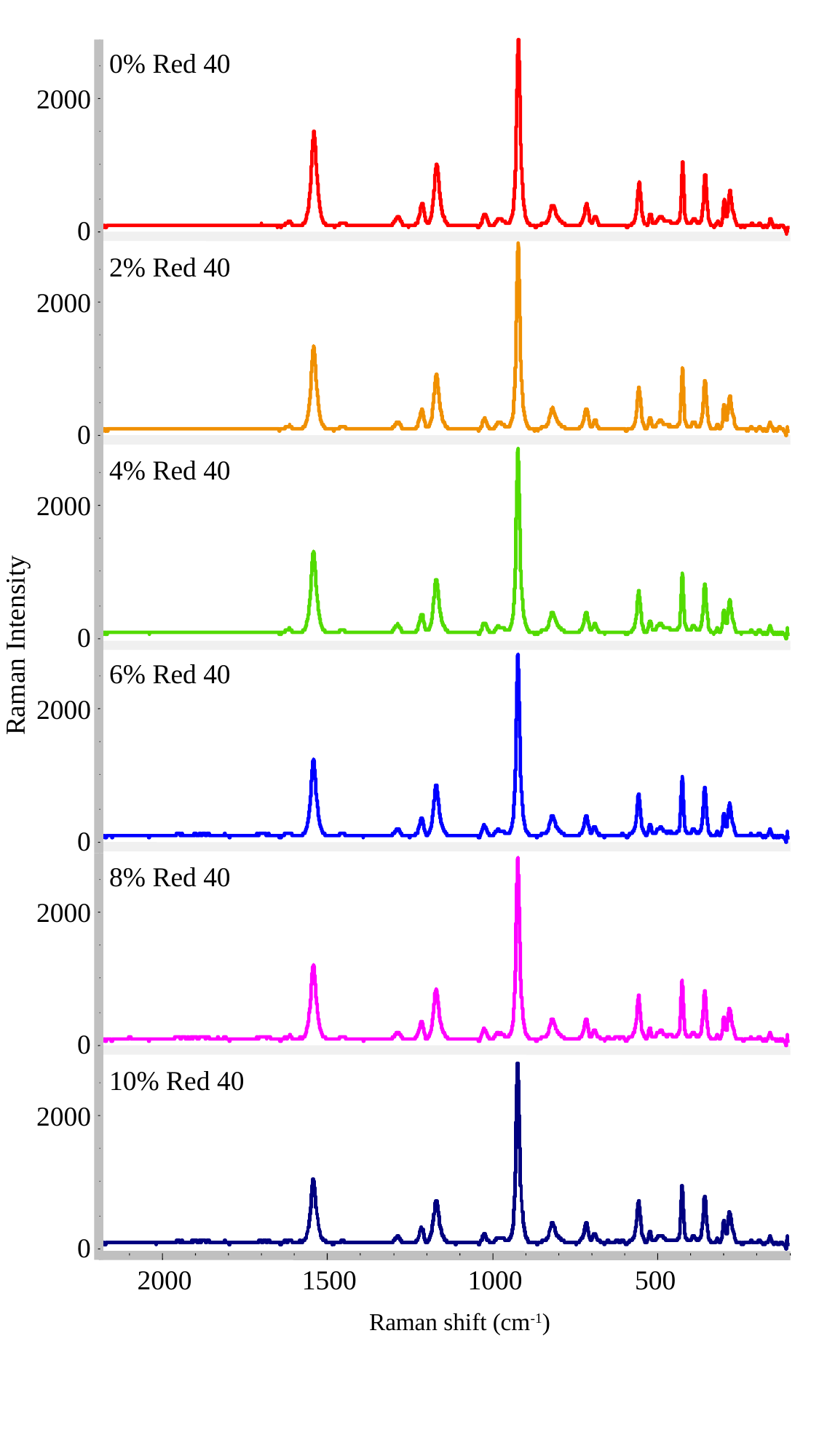

0% Red 40
 2000
 0
2% Red 40
 2000
 0
4% Red 40
 2000
 0
Raman Intensity
6% Red 40
 2000
 0
8% Red 40
 2000
 0
10% Red 40
 2000
 0
 2000
 1500
 1000
 500
Raman shift (cm-1)

## Slide 6
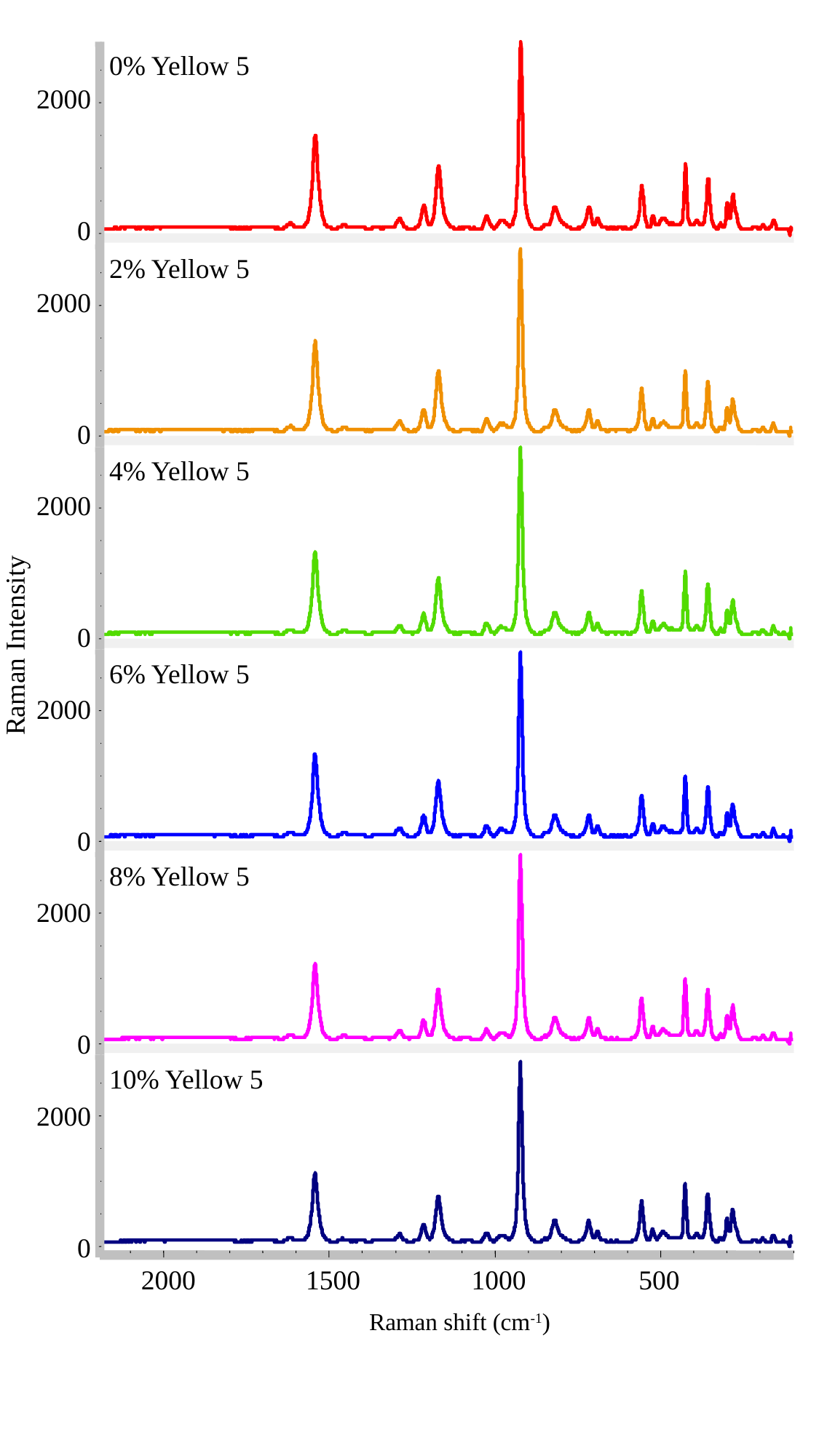

0% Yellow 5
 2000
 0
2% Yellow 5
 2000
 0
4% Yellow 5
 2000
 0
Raman Intensity
6% Yellow 5
 2000
 0
8% Yellow 5
 2000
 0
10% Yellow 5
 2000
 0
 2000
 1500
 1000
 500
Raman shift (cm-1)

## Slide 7
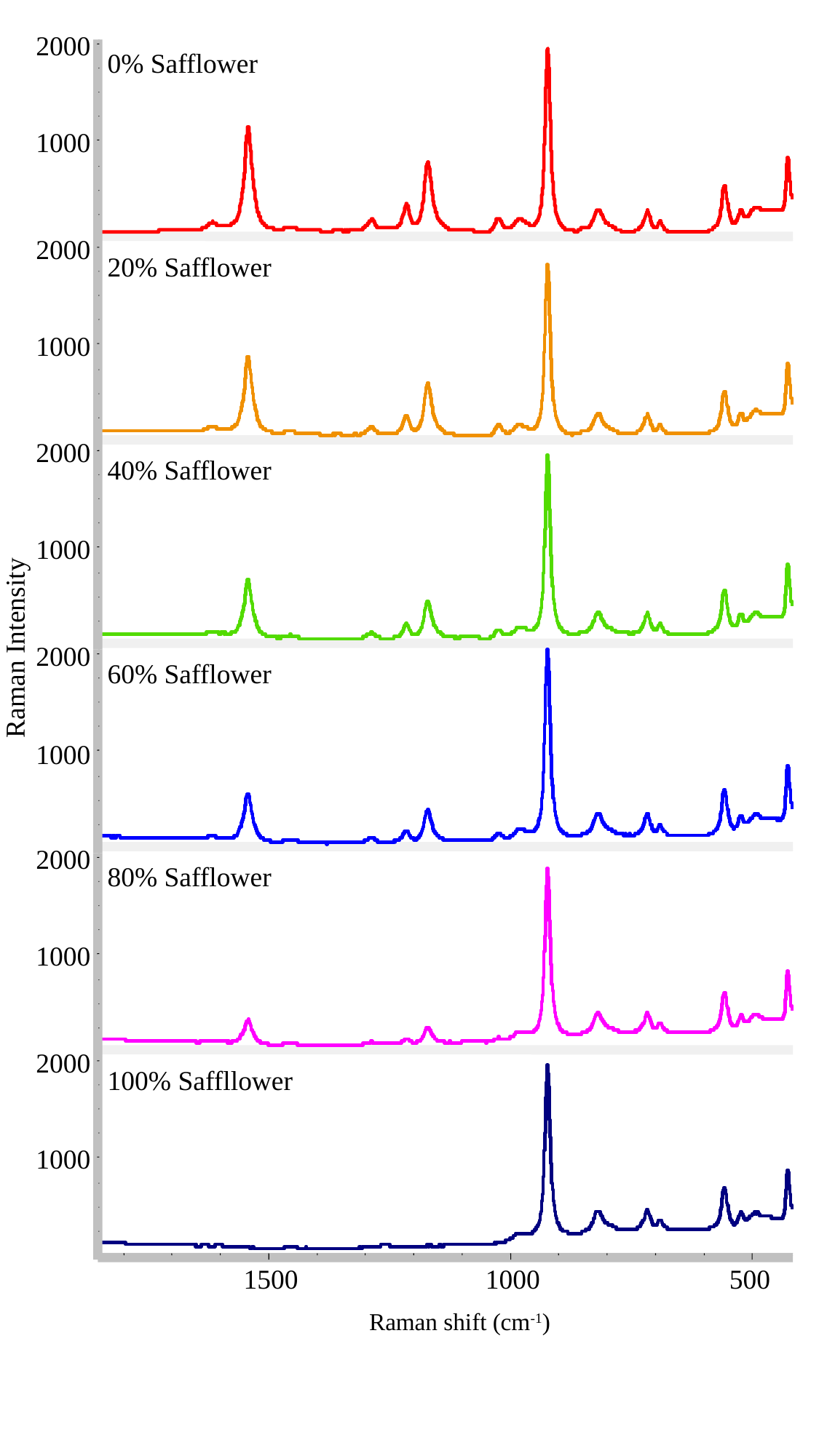

2000
0% Safflower
 1000
 2000
20% Safflower
 1000
 2000
40% Safflower
 1000
Raman Intensity
 2000
60% Safflower
 1000
 2000
80% Safflower
 1000
 2000
100% Saffllower
 1000
 1500
 1000
 500
Raman shift (cm-1)

## Slide 8
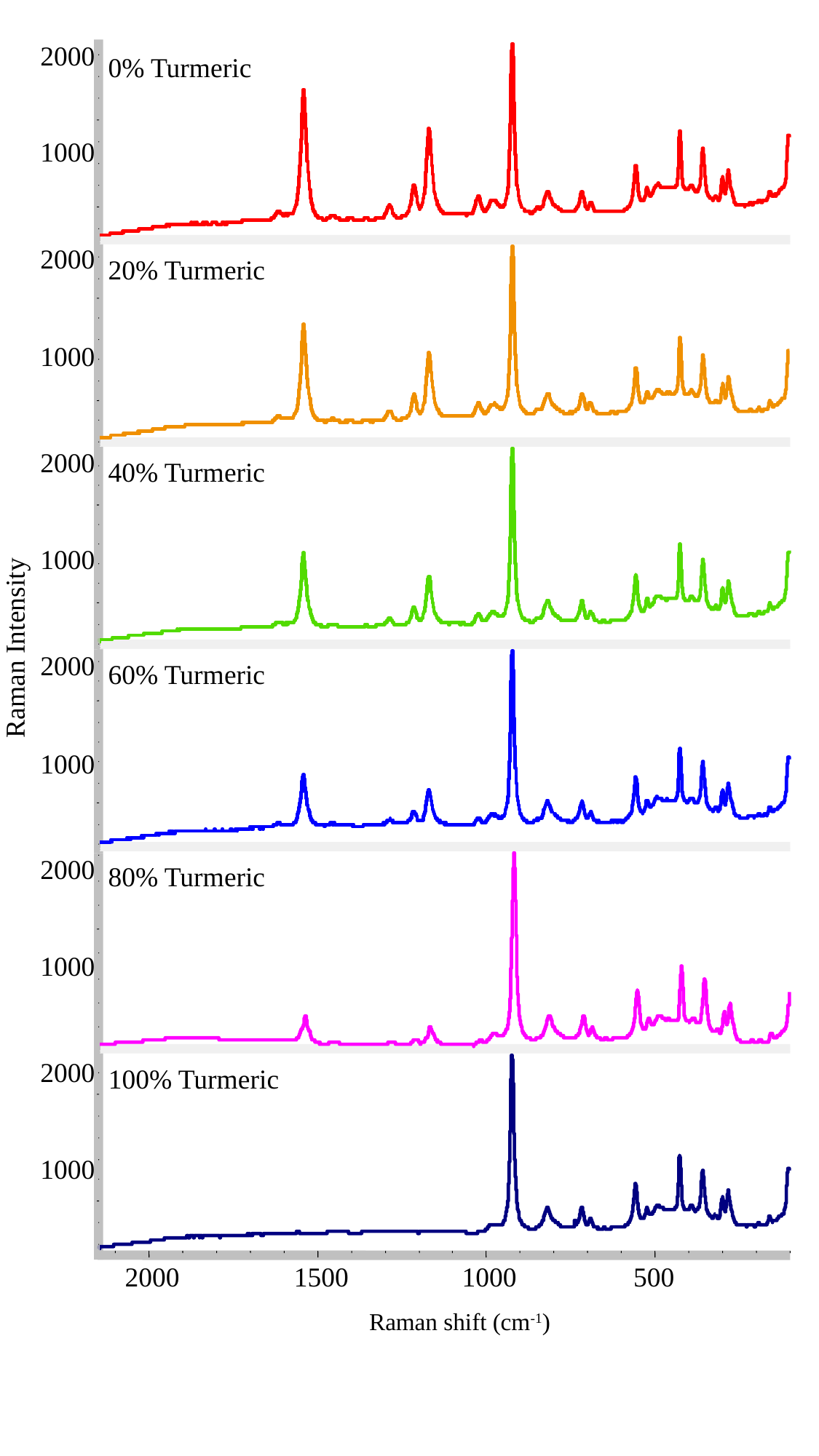

2000
0% Turmeric
 1000
 2000
20% Turmeric
 1000
 2000
40% Turmeric
 1000
Raman Intensity
 2000
60% Turmeric
 1000
 2000
80% Turmeric
 1000
 2000
100% Turmeric
 1000
 2000
 1500
 1000
 500
Raman shift (cm-1)
